# Reliability of cardiac computed tomography examination in cardiac pathology – a case series and literature review

**DOI:** 10.25122/jml-2023-0268

**Published:** 2023-07

**Authors:** Adriana Sorina Capisizu, Silviu Stanciu, Dragoș Cuzino

**Affiliations:** 1 Department of Radiology and Imagistic Medicine 1, Faculty of Medicine, Carol Davila University of Medicine and Pharmacy, Bucharest, Romania; 2 Department of Cardiology, Faculty of Medicine, Carol Davila University of Medicine and Pharmacy, Bucharest, Romania; 3 Center for Cardiovascular Diseases, Laboratory of Noninvasive Cardiovascular Functional Explorations, Dr. Carol Davila Central Military Emergency University Hospital, Bucharest, Romania; 4 Clinical Radiology-Medical Imaging Center, Medical Imaging Radiology Laboratory II, Dr. Carol Davila Central Military Emergency University Hospital, Bucharest, Romania

**Keywords:** cardiac computed tomography, cardiac case report

## Abstract

Due to the technological progress in the last decade, the medical practice of cardiology relies more and more on the information provided by cardiac computed tomography. Current guidelines propose coronary computed angiography as a first-line anatomic investigation in patients with low and intermediate probability of coronary atherosclerosis. At the same time, anatomical information is obtained about the rest of the cardiac structures and about cardiac function. The present work aimed to present the contribution of cardiac computed tomography in the morphological and functional characterization of patients with complex cardiac pathology. Three cases are presented in which the information provided by cardiac computed tomography contributed significantly to the diagnosis. The cases were cardiac pathologies such as ventricular apical aneurysm, aortic prosthetic thrombosis, and a cardiac malformation with transposition of great arteries. In addition to highlighting the role of cardiac computed tomography in diagnosing cardiac pathology, a brief review of cardiac computed tomography indications, inclusion criteria, patient safety, basic techniques, and the main protocols and types of post-processing used in practice is provided. This article provides an overview of the applications of cardiac computed tomography, highlighting its role within clinical practice, particularly in the characterization of complex cardiac pathologies.

## INTRODUCTION

Over the past decade, substantial technological advancements in computed tomography (CT) have led to an increasing reliance on the information provided by cardiac CT examinations in worldwide medical practice. [1]. In 2019, the European Society of Cardiology (ESC) established coronary computed tomography angiography (CCTA) as the primary anatomical investigation in patients with coronary atherosclerotic disease [2].

At the same time, anatomical information about cardiac cavities, thoracic aorta, cardiac venous structures, and pulmonary structures included in the examination field and functional information about volumetry and ventricular function can be obtained [1].

Indications for coronary CT angiography are numerous and part of the complex cardiovascular evaluation. Examination accuracy and image quality largely depend on knowledge of the patient selection procedure.

CCTA indications are recommended by the ESC guidelines for different categories of patients depending on the symptomatology:

- the diagnosis and evaluation of symptomatic patients with low and medium probability of atherosclerotic coronary disease, which was recently updated as a class I indication [2, 3];
- evaluation of patients with new stable angina [3, 4];
- patients with inconclusive electrocardiographic (ECG) or laboratory results;
- patients with uncertain stress tests;
- evaluation of coronary grafts and intrastent restenosis [1, 2, 5].

Patient selection for coronary CT angiographic investigation has various inclusion and exclusion criteria and is the basis of CT image quality.

- Inclusion criteria of patients are heart rate below 65 beats/minute and ability to maintain apnea [6].
- Exclusion criteria include patients with a heart rate over 70 beats/minute, known allergies to iodinated contrast material, pregnancy, and patients with renal failure with serum creatinine values over 120 mmol/l [1]. The broadening of the inclusion criteria implies hydration and dialysis of patients with low renal function, respectively, and the administration of steroid premedication in patients with allergies [6].

The extensive use of CCTA has led to the diagnosis of complex cardiac pathologies, both as a complementary and unique diagnostic method. Thus, CCTA is successfully used to diagnose cardiac apex aneurysms where routine cardiac evaluation by cardiac ultrasound may have limited contribution [7]. A significant example is illustrated by a presented case involving a patient with a history of stroke and no known cardiovascular risk factors. Clinical valve thrombosis after transcatheter aortic valve implantation (TAVI) that associates thrombus formation with increased transvalvular gradient and the production of heart failure symptoms is rare, with a prevalence of 1.2% [8]. Cardiac ultrasound remains the primary investigation in evaluating prosthetic aortic valve thrombosis, but given technological progress and protocol adaptation, CCTA provides important complementary information that increases diagnostic accuracy [9, 10]. Corrected congenital transposition of the great arteries (CCTGA) is a rare cardiac malformation with an incidence of less than 1%, often asymptomatic, with varied morphologic presentation, some requiring surgical intervention. CCTA can help characterize the cardiac structure report and further appreciate anatomical variants [11, 12].

## CASE PRESENTATION

### Case 1

A 54-year-old female patient was admitted to the cardiology clinic with recurrent symptoms of angina complicated by C5-C6 cervical spondylosis. The patient had a history of stroke in 2019 and constrictive pericarditis, treated surgically in 2012.

**Figure 1:**
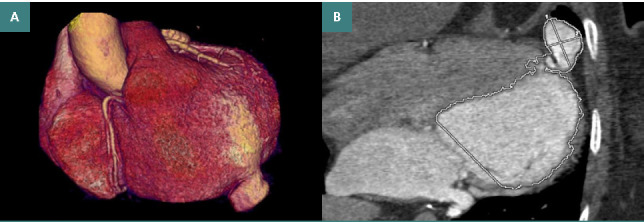
Ventricular aneurysm (A) 3D volume rendering; (B) multiphase oblique long axis.

- Clinical examination: BP=120/94 mmHg and pulse= 75 beats/minute.
- Standard ECG: sinus rhythm, AV=75 beats/minute, without terminal phase repolarization changes.
- Cardiac ultrasound: VSTD=45 mm, FEVs=60%, SIV walls=12 mm, no abnormalities of segmental kinetics.
- Laboratory: Total lipids=1041.0 mg/dl (range 350.00 – 800.00 mg/dl), Total Bilirubin=1.39 mg/dl (range 0.30 – 1.20 mg/dl), Indirect Bilirubin=1.21 mg/dl (range 0.1 – 1.00 mg/dl). The rest of the laboratory values were within normal range.
- CCTA: The patient received intravenous beta-blockers at the start of the examination to achieve a low heart rate of <70 beats/minute. The images revealed a pseudoaneurysm of the cardiac apex, with maximum dimensions of 17 mm latero-lateral, 24 mm antero-posterior, and 14 mm craniocaudal, with persistence of the contrast substance (Fig.1 A, B).

*Diagnostic*: Complicated angina, ventricular cardiac aneurysm.

Discharge recommendations included close collaboration between the neurologist-cardiologist team to determine anticoagulant treatment decisions and the cardiovascular surgery service for decisions regarding surgical closure of the apical pseudoaneurysm.

### Case 2

A 75-year-old female patient with a bioprosthetic aortic valve Medtronic No. 21 for severe aortic stenosis, inserted two years earlier, was admitted for reevaluation. Other pathological antecedents of the patient were grade III hypertension, class II obesity, dyslipidemia in treatment, and trans-hiatal gastric hernia.

**Figure 2:**
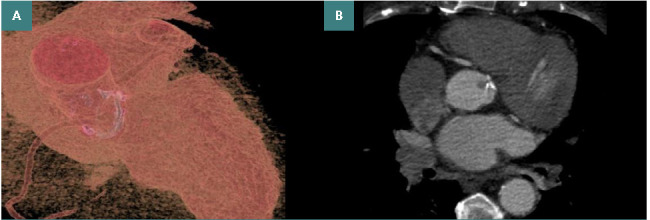
Aortic valve prosthesis (A) 3D volume rendering; (B) Axial Ph 75%, hypodense aortic valve thrombus.

- Patient history: TTE ultrasounds performed 5 months earlier revealed dysfunctional prosthesis, with inexactly evaluable morphology, increased transvalvular gradient, mean-maximum LV-Ao gradient in the range of 42-71 mmHg, calculated AVA of 0.8 cm^2^, intraprosthetic regurgitation grade II and non-dilated left ventricle with preserved function. The TEE ultrasound performed 1.5 months prior revealed a dysfunctional aortic bioprosthesis due to thrombosis and increased transvalvular gradient. Consequently, it was decided to increase the dose of AVK anticoagulant while maintaining the INR at approximately 3.
- Present clinical examination: hemodynamic and respiratory stable patient, BP=165/85 mmHg.
- CCTA: prosthetic aortic valve complication, identification of a 7/8 mm hypodense, low-attenuating, non-enhancing structure, <90 Hounsfield units, on the anterior plane of the aortic prosthesis attached to the right leaflet, suggestive of valve thrombosis (Fig. 2 A, B).

*Diagnostic*: Dysfunctional aortic valve prosthesis with complications, aortic valve thrombosis.

As part of the initial discharge instructions, it was recommended that the patient initiated anticoagulant treatment using vitamin K antagonists, maintaining a therapeutic INR of 3 and INR dosing every two weeks, and being in the attention of the cardiovascular surgery service for transcatheter aortic valve implantation.

### Case 3

A 66-year-old female patient with known corrected great vessel transposition diagnosed by cardiac ultrasound, apical obstructive hypertrophic cardiomyopathy (apical gradient 114 mmHg), mild mitral regurgitation, history of occasional self-resolving chest discomfort, and abnormal ECG was admitted for CT coronary angiographic evaluation. There were no other significant cardiovascular risk factors.

**Figure 3:**
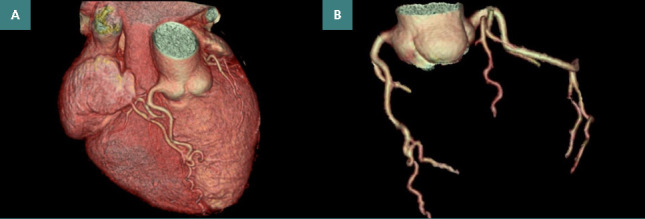
Congenital malformation with corrected transposition of the great vessels (A) multiphase 3D volume rendering revealing aorta to the left and anterior to the pulmonary artery; (B) 3D volume rendering ph 75% coronary tree anatomy.

- Present clinical examination: hemodynamic and respiratory stable patient, asymptomatic during clinical examination, no signs of systemic or pulmonary congestion.
- Standard resting ECG: sinus rhythm, QS complexes in DIII and aVF, negative T waves in aVL.
- Laboratory: values within normal range.
- CCTA: defined anatomy and confirmed CCTGA with S, L, L type (situs solitus of the atria with L-loop ventricles and L-transposed great arteries). Comprehensive morphological imaging showed parallel vessels emerging from the heart, with the aorta to the left and anterior to the pulmonary artery. The aorta was connected to the morphological right ventricle (RV), located on the left. The pulmonary artery was connected to the morphological left ventricle (LV). The connections between the ventricles and the atria were discordant, with the left atrium connected to the morphological RV and the right atrium connected to the morphological LV. An abnormal coronary artery anatomy was also observed, characterized by unusual rotation of the coronary cusps. The noncoronary cusp was anterior and to the left, while the right coronary cusp, in relation to the patient, led to a coronary artery perfusing the anterior interventricular groove. Furthermore, the posterior-facing coronary cusp gave rise to a coronary artery that bifurcated into a branch descending along the morphological right ventricle and another descending to the left within the atrioventricular groove. No significant coronary artery stenosis was found. Coronary arteries did not show atheromatous infiltration (Fig. 3 A, B).
- Coronary calcium score=0.
- Functional assessment of the ventricles showed FE Systemic ventricle=71 and FE Pulmonary ventricle=42. No other significant intracardiac abnormalities or complications were associated.
- Holter ECG/24h: sinus rhythm, average AV 82/min, AV min 60/min, AV max 130/min, isolated ventricular and supraventricular extrasystoles, two supraventricular triplets, no pathological pauses.

*Diagnostic*: Congenital malformation with corrected transposition of the great vessels

As part of the discharge recommendations, the patient was advised to engage in physical exertion within tolerated limits. Additionally, the patient was encouraged to stay under the care of the cardiology service for ongoing function monitoring and return for cardiac magnetic resonance imaging.

## DISCUSSION

### Indications and performance of CCTA in cardiac pathology

CCTA has been successfully used to diagnose an aneurysmal lesion of the cardiac apex in a patient with a history of ischemic stroke, in whom cardiac ultrasound had limited input in evaluating the cardiac apical region. Moreover, it was possible to evaluate the size of the aneurysmal dilatation in three planes and to analyze the opacification with the contrast agent. Regarding the thrombotic complication after aortic valve replacement, CCTA is shown to bring a wide variety of information in the diagnosis of valve thrombosis and the mechanism of functional valve dysfunction, especially by identifying hypoattenuating leaflet thrombosis and pannus formation [7, 13-16]. In general, CCTGA is a cardiac malformation that, despite discordance between atria and ventricles, between ventricles and arteries, is associated with physiological blood circulation without arterio-venous shunts [11]. In any case, it is important to analyze the anatomy and the variations that may occur and complicate CCTGA. In this regard, CCTA has a superior contribution to the 3D volumetric visualization of cardiac structures and their report.

### Basic technical and performance concepts of CCTA

The most important components of the computed tomography system are the X-ray tube and the detector system, and the working principle of CT is to rotate the X-ray tube assembly with the detector system around the patient to obtain sections covering the scanned area [1, 6].

The biggest challenge is to obtain artifact-free images of rapidly moving heart, for which high temporal and spatial resolution heart-synchronized systems are used, with the institutional hardware requirement being CT machines with at least 64 slices/rotation [1, 5, 6].

CT systems are divided into two categories based on the mode of image acquisition. One is the conventional sequential mode of acquiring each section followed by moving the CT table, and the other is the modern spiral mode with the achievement of continuous spiral acquisition of the tube-detector assembly [6].

Given these two types of action of CT systems, there are two broad cardiac acquisition protocols. The first is the retrospective spiral with ECG monitoring with continuous data acquisition throughout the cardiac cycle, and the second is prospective at a specific preselected cardiac cycle time. Each protocol has specific advantages and indications.

Retrospective acquisitions offer flexibility; the acquired data are complete, and image reconstruction can occur at any point in the cardiac cycle. The cardiac cycle consisting of systole and diastole is detected by the ECG trace showing the typical wave sequence: P wave corresponding to atrial contraction, QRS complex corresponding to ventricular contraction, and T wave corresponding to ventricular repolarization. The retrospective cardiac reconstruction window is usually positioned in the mid- and end-diastolic phase, just before the P wave, at about 75% of the R-R interval, where there are usually fewer artifacts from cardiac motion. A second window of limited cardiac motion is at the end of systole, approximately 35% of the R-R interval [1, 6].

Prospective acquisitions are conducted during the end-diastolic phase, with current modulation in the systolic phase, which offers the advantage of reduced radiation doses. The resulting images are more susceptible to artifacts due to rhythm irregularities and involve less post-processing capability to remove them. It also involves advanced systems with detectors wide enough to cover cardiac volume, scanning 120 mm - 150 mm in 4 - 5 steps. The technique applies only to selected patients with good heart rate control below 60-65 beats/minute, and a regular heart rate can be achieved [1].

The best current CT systems include the maximum parameters: rotation times 250 ms, temporal resolution 135 ms, 66 ms for dual source, obtaining 320 sections/rotation, and spatial resolution below 0.5 mm isotopic, which allows visualization of details in oblique reconstructions [6].

The most innovative CCTA techniques target the functional aspects of coronary lesions, with computational fluid dynamics and stress CT perfusion with adenosine [3, 17]. The widespread introduction of these functional assessments into the CCTA protocol aims to transform it into a unique stop in the patient investigation pathway that simultaneously establishes anatomical and functional information [3]. One of the most recent concepts in CCTA postprocessing is the assessment of peri coronary fat to evaluate the pathophysiological processes and inflammation at the coronary level that could cause myocardial infarction [3, 18].

#### Patient preparation and safety information

Patient preparation is very useful because the quality of CT coronary angiography images depends on it. This preparation includes routine premedication with a β-blocker administered orally under the direction of the attending physician to decrease the heart rate. In some instances, intravenous supplementation during the examination may also be considered. Caution is recommended in the contraindications of beta-blockers: heart failure, heart blocks, aortic stenosis, and bronchial asthma. At the beginning of the examination, sublingual nitroglycerin is administered to control vascular tone and dilate the coronary arteries [1, 6, 19]. In addition to the usual contraindications to nitroglycerin (allergy to nitroglycerin, early myocardial infarction) and precautions (patients with critical aortic stenosis), reactions can occur in the context of the administration of beta-blockers and nitroglycerin, the most common of which is a decrease in systolic blood pressure >20mmHg. Studies have shown a low incidence (1.2%) of adverse reactions during CCTA but higher in older men with a high atherosclerosis burden. Reducing the dose of nitroglycerin to 0.4 mg versus 0.8 mg reduced the rate of decrease in blood pressure [19].

The safety of patients undergoing CCTA is continuously evaluated. Possible adverse reactions include anaphylactic reactions, vasovagal symptoms, and extravasation of contrast material and serum, but overall, CCTA is a safe examination in which adverse reactions are rare, even in the context of administration of beta-blockers [19].

#### Imaging protocol for CCTA investigation

The CCTA investigation procedure includes two scanning steps: the first scan is native to assess the Agatston score or coronary calcium score, and the second is a post-contrast scan after intravenous iodinated contrast agent [1]. The intravenous injection of contrast agent is carried out at a high flow rate of 4-5 ml/sec, utilizing a concentration of 300-400 mg/ml and a volume between 40 ml and 100 ml. [1, 6]. Consequently, there is a narrow timeframe for capturing full-contrast images due to the variable transit time – ranging from 20 to 40 seconds – from injection at the elbow crease to the heart, depending on the cardiac output of each patient [6]. In current practice, injection protocols include the administration of saline solution bolus chaser that will reduce contrast dose and artefacts in the right atrium and superior vena cava and enhance contrast visualization of the coronary arteries, particularly the right coronary [1, 6].

The radiation exposure to patients during cardiac CT examinations ranges from 1 to 10 mSv, aligning with the annual dose limit of 1 mSv for the general population from natural environmental sources, excluding medical exposures [6, 20].

### Types of imaging evaluation with CCTA

Image post-processing is interactive, involving dedicated software applications, and has a major role in integrating the acquired information [1].

- For experienced examiners, a first evaluation of the source images in the axial plane is essential, considering that the subsequent post-processing includes partial data from the initial acquisition [6]. Both coronary structures and the rest of the heart structures are evaluated; coronary stenoses are evaluated in a semiquantitative manner involving segments larger than 1.5 mm in diameter, generally using the American Heart Association segmentation [1].
- Multiplanar reconstructions (MPR) constitute the subsequent assessment phase involving 2D images. Planar and curved reconstructions of the coronary arteries can be obtained, improving especially the tortuous vascular trajectories, with the possibility of rotation around the axis and obtaining axial sections in the plane of the vessel. Compositional analysis of plaques according to attenuation is performed, and the calcified, fibrous, or lipid-rich structure is analyzed. Lipid plaques with an attenuation of less than 30 UH are associated with an increased risk of rupture [1, 5, 6, 21].
- Maximum Intensity Projections (MIP) are 2D reconstructions that display only the maximum intensity pixels and produce images similar to conventional angiography that can be rotated 360° with good visualization of the vascular lumen [1, 5, 6].
- 3D volumetric reconstructions (VR) use various degrees of transparency of structures depending on their CT density value, thus creating depth and perceiving the relationship of structures in space. The opacity level varies, and color codes can be assigned to individualize cardiac structures. These are images used for general visualization of the coronary tree and cardiac structures [1, 2, 6].

#### Types of measurements analyzed by CCTA

Image post-processing is interactive, involving dedicated software applications, and has a major role in integrating the acquired information [1].

- For experienced examiners, a first evaluation of the source images in the axial plane is essential, considering that the subsequent post-processing includes partial data from the initial acquisition [6]. Both coronary structures and the rest of the heart structures are evaluated; coronary stenoses are evaluated in a semiquantitative manner involving segments larger than 1.5 mm in diameter, generally using the American Heart Association segmentation [1].
- Multiplanar reconstructions (MPR) constitute the subsequent assessment phase involving 2D images. Planar and curved reconstructions of the coronary arteries can be obtained, improving especially the tortuous vascular trajectories, with the possibility of rotation around the axis and obtaining axial sections in the plane of the vessel. Compositional analysis of plaques according to attenuation is performed, and the calcified, fibrous, or lipid-rich structure is analyzed. Lipid plaques with an attenuation of less than 30 UH are associated with an increased risk of rupture [1, 5, 6, 21].
- Maximum Intensity Projections (MIP) are 2D reconstructions that display only the maximum intensity pixels and produce images similar to conventional angiography that can be rotated 360° with good visualization of the vascular lumen [1, 5, 6].
- 3D volumetric reconstructions (VR) use various degrees of transparency of structures depending on their CT density value, thus creating depth and perceiving the relationship of structures in space. The opacity level varies, and color codes can be assigned to individualize cardiac structures. These are images used for general visualization of the coronary tree and cardiac structures [1, 2, 6].

#### Types of measurements analyzed by CCTA

The measurements that can be analyzed by CCTA are both structural and functional.

- The structures of the heart chambers are highlighted by standard cardiac visualization planes, similar to cardiac ultrasound, which can be obtained by post-processing: two chambers with the view of the atrium and left ventricle, three chambers with the view of the atrium and the left ventricle and the root of the aorta, four chambers with visualization of the left and right heart cavities and atrioventricular valves, short axis.
- The venous vascular structures that can be analyzed are the venous system returning to the right atrium consisting of the superior and inferior vena cava, the coronary sinus, and the pulmonary veins. The ejection tract of the right ventricle with the trunk of the pulmonary artery is also visualized with the possibility of dimensional measurements.
- The proximal aorta is an essential assessment in TAVI procedures, measuring the aortic root in systole and diastole. There are dedicated protocols, and the essential elements that must be included in the final report are the diameter of the aortic annulus, the sinus of Valsalva, and the sino-tubular junction.
- The extracardiac structures - mediastinum and lungs included in the examination field are analyzed, and possible changes are mentioned in the final report.
- Ventricular volume and function measurements are performed semi-automatically for the left ventricle in imaging protocols throughout the cardiac cycle [6].
- The Agatston calcium score - is a standardized semi-automated method for assessing the coronary calcium score.

### Cardiac CCTA limits

- Low spatial resolution relative to small-diameter coronary vessels remains a limitation in evaluating coronary stenoses.
- The presence of poor image quality and artifacts leads to overestimation of stenoses.
- Extensive coronary calcifications generate “blooming” artifacts.
- Higher accuracy and better-quality images were demonstrated in patients with low and intermediate probability of atherosclerotic coronary artery disease compared to patients with higher probability.
- In patients with coronary grafts, coronary CT angiography has limitations in evaluating the native arteries but has a good ability to appreciate the presence of stenoses at the level of the grafts.
- Evaluation of stents is difficult due to the metal artifacts generated by them and highly depends on the quality of the images obtained, the caliber of the vessel, and the stent. The evaluation is reliable for stents with a diameter of 3.0 mm and above. [1, 6].

## CONCLUSION

Coronary CT angiography is a modern and particularly useful investigation for cardiac patients. The advancements in technology have significantly enhanced the performance of CCTA, yielding high-quality images that not only aid in diagnosing a range of cardiac pathologies extending beyond coronary disease but also play a pivotal role in shaping patient management strategies.

## Data Availability

Further data is available from the corresponding author upon reasonable request.
